# Transovum Transmission of Trypanosomatid Cysts in the Milkweed Bug, *Oncopeltus fasciatus*

**DOI:** 10.1371/journal.pone.0108746

**Published:** 2014-09-26

**Authors:** Felipe de Almeida Dias, Luiz Ricardo da Costa Vasconcellos, Alexandre Romeiro, Marcia Attias, Thais Cristina Souto-Padrón, Angela Hampshire Lopes

**Affiliations:** 1 Instituto de Bioquímica Médica Leopoldo de Meis, Programa de Biologia Molecular e Biotecnologia, Universidade Federal do Rio de Janeiro, Rio de Janeiro, RJ, Brazil; 2 Instituto de Microbiologia Paulo de Góes, Universidade Federal do Rio de Janeiro, Rio de Janeiro, RJ, Brazil; 3 Instituto de Biofísica Carlos Chagas Filho, Universidade Federal do Rio de Janeiro, Rio de Janeiro, RJ, Brazil; International Atomic Energy Agency, Austria

## Abstract

*Leptomonas wallacei* is a trypanosomatid that develops promastigotes and cystic forms in the gut of the hemipteran insect *Oncopeltus fasciatus*. Insect trypanosomatids are thought to be solely transmitted from one host to another through the ingestion of parasite-contaminated feces. However, here we show that *L. wallacei* cysts present on the eggshells of eggs laid by *O. fasciatus* can also act as infective forms that are transmitted to the insect offspring. Newly hatched *O. faciatus* nymphs are parasite-free, but some of them become contaminated with *L. wallacei* after feeding on eggshell remnants. The present study is the first report of transovum transmission of a trypanosomatid, a process that may have a relevant role in parasite’s within-host population dynamics.

## Introduction

The family Trypanosomatidae is known by the severe human diseases caused by some of its species, which kill several thousand people yearly. Roughly 37 million people worldwide are infected with *Trypanosoma brucei* (African sleeping sickness), *Trypanosoma cruzi* (Chagas disease) and *Leishmania* species (different forms of leishmaniasis) [1]. Trypanosomatids are evolutionarily successful organisms, parasitizing a broad range of invertebrates, vertebrates, plants [2] and even other protozoans [3]. Sixty percent of trypanosomatid genera comprise of monoxenous insect parasites and the remainder forty percent comprise mostly of vertebrate parasites transmitted by insects [4]. Nevertheless, there are very few reports on the life cycles of insect trypanosomatids [5]. *Oncopeltus fasciatus* is a natural host of *Leptomonas wallacei*, which is a gut-restricted insect trypanosomatid [6]–[8]. *L. wallacei* develops free-swimming promastigotes, mainly in the midgut, and promastigotes attached to the intestine wall, mostly in the hindgut [6]–[8]. Promastigotes often display encysting stages (straphangers) adhered to the flagellum; these cystic resistant forms are frequently found in clusters of two to four. Free, mature cysts can also be found in the lumen of the intestinal tract, especially in the hindgut [6]–[8].

The milkweed bug *O. fasciatus* is a hemipteran insect, which has been an important model for classical studies on embryogenesis [9]–[11]. More recently, *O. fasciatus* has become a laboratory model for reports on molecular development of insects [12], [13], transcriptomes [14], [15], as well as several aspects of the interaction of these insects with their natural [7], [8] or experimental trypanosomatid parasites [16]–[19].

There are basically two forms of transmission of endoparasites among insects: vertical, when the parasite is transferred from the parent to its progeny, and horizontal, when the transfer occurs between two individuals, either host to host, or host to environment and then to host [20]–[22]. The vertical transmission is subdivided into two forms: transovarial and transovum, in which the parasites are present within the eggs or on the eggshells, respectively [20], [23], [24]. In the latter scenario, the parasites are acquired by the newly hatched nymphs or larvae by feeding on eggshell remnants [24]. Transmission and virulence of parasites are decisive factors that determine host-parasite relationships. Vertical transmission demands precise integration of the parasite with the host biological functions and may be an exceptional predictor to ecological host specificity [20]–[22]. In migratory insects, such as *O. fasciatus* [11], vertical transmission may be crucial to the persistence of parasites in host populations [25].

The vertical transmission of trypanosomatids among invertebrate hosts has been proposed but so far not confirmed [26], [27]. The main goal of the present study was to experimentally show the existence of transovum transmission of *L. wallacei* by *O. fasciatus*. For the first time, we demonstrated that trypanosomatid cysts may be vertically transmitted from insect females to their offspring through contamination of the egg surface with feces that contains cysts, and subsequent ingestion of these feces by newly hatched nymphs. Therefore, we believe that the differentiation of *L. wallacei* promastigotes to cystic forms and the vertical transmission of parasites to the host offspring may be connected and working together for the success of parasitism.

## Materials and Methods

### *Oncopeltus fasciatus* colony

A colony of *O. fasciatus* naturally infected with *L. wallacei* [7] was established and maintained in our laboratory under a 12 h light/dark cycle at 28°C with 70–80% relative humidity, as previously described [6], [18].

### Establishment of a parasite-free colony

In order to obtain a parasite-free colony, eggs collected from the infected colony were submitted to surface asepsis. The asepsis was performed by treatment of the eggs with 2% sodium hypochlorite for 5 min, which was followed by washing the eggs in distilled sterile water and drying on sterile filter paper. After asepsis, the eggs were kept in sterile plastic containers and the newly-hatched insects maintained in the same conditions described for the parental colony. In order to avoid recontamination of the parasite-free colony, these insects have been kept in a different, isolated, room from the parental colony. In order to validate the absence of *L. wallacei* in the parasite-free colony, samples of five insects collected at random have been weekly checked for the presence of *L. wallacei* in their guts by optical microscopy and PCR.

### *Leptomonas wallacei* culture

*L. wallacei* was grown in Warren modified medium (37.0 g/l brain infusion hearth, 10.0 µg/l folic acid and 1.0 mg/l hemin) at 28°C, supplemented with 10% fetal calf serum. In the logarithmic growth phase, the parasites were washed three times with phosphate-buffered saline, pH 7.2 (PBS) and harvested by centrifugation at 5.000×g for 10 min at 4°C for DNA extraction.

### Analysis of *L. wallacei* infection during *O. fasciatus* life cycle

To establish the time course of *O. fasciatus* infection by *L. wallacei,* 450 *O. fasciatus* eggs were collected from the infected colony, separated in three groups of 150 eggs and kept in separated sterile plastic containers for eclosion. In the third-, fourth- and fifth instar nymphs and in adults, 30 insects were randomly collected from each one of the four groups and dissected in PBS. The guts were then extracted for analysis of infection by optical microscopy in a Zeiss Axioplan 2 light microscope (Oberkochen, Germany) equipped with a Color View XS digital video camera. Insects with at least one mobile flagellate in their alimentary tract were considered infected. Parasite detection was conducted through the analysis of optical micrographs taken from Giemsa-stained gut contents of the aforementioned insects. Promastigotes, encysting stages (straphangers) and free cystic forms were identified as described [6], [28]. Some adult females were transferred to another plastic container for fresh feces and egg collection. The fresh and dried feces, the eggs and the gut of these adult insects were processed for scanning electron microscopy (SEM).

### Transmission of *L. wallacei* via eggshells

Eggs collected from the infected colony were mechanically broken, mixed with sterile sunflower seeds and offered as the only source of food to 200 *L. wallacei*-free adult insects, in a sterile plastic pitcher. After one week, 20 insects were dissected in Petri dishes containing sterile PBS and the guts extracted for analysis of infection by SEM or to DNA extraction. DNA samples were PCR-amplified for parasite detection.

### Detection of parasite infection by PCR

*L. wallacei* promastigotes grown in axenic culture medium, insect guts or eggs were homogenized in 500 µl lysis buffer (Tris-HCl (pH 7.6), 0.1 M NaCl, 10 mM EDTA, 0.5% SDS and 300 µg/ml proteinase K), incubated at 52°C for 1 h and submitted twice to extraction with phenol:chloroform:isoamyl alcohol (25∶24∶1,v/v). The samples were centrifuged at 5,000×*g* for 10 min. After extraction, total DNA was precipitated from 200 µl-aliquots of the aqueous phase by centrifugation at 5.000×*g* for 10 min at room temperature, after the addition of 25 µl 3 M sodium acetate and 475 µl absolute ethanol. The pellet of DNA was dried and resuspended in distilled water. Primers specific for *O. fasciatus* were designed by our group using as target the sequence of *O. fasciatus* 16S rRNA gene (GenBank accession number AY252660.1). Since there are no *L. wallacei* gene sequences deposited in the Gen Bank, we designed specific primers for a conserved region present in all of 25 trypanosomatid 18S rRNA gene sequences obtained from GenBank database. The trypanosomatid sequence are the following: GenBank accession numbers FJ968532.1; GQ332362.1; GQ332358.1; GQ332355.1; GQ332363.1; GQ332361.1; GQ332360.1; GQ332354.1; GQ332359.1; DQ383648.1; DQ910924.1; EU079129.1; AF153039.1; AF153036.1; EU021240.1; DQ910925.1; DQ910923.1; EU079128.1; EF546786.1; AF153043.2; U01013.1; EU267074.1; U01016.1; FJ968531.1; and DQ383649.1. The sequences of the primers designed respectively for *O. fasciatus* and *L. wallacei* are as follows: F-Lw 5′-CTTTTGGTCGGTGGAGTGAT-3′and R-Lw 5′-GGACGTAATCGGCACAGTTT-3′; F-Of 5′-CAAAATTTGGTTGGGGTGAC-3′ and R-Of 5′-ATCGAGGGTCGCAAACTCTT-3′. The amplification reactions were performed in a final volume of 10 µl. Each reaction was performed with 50 ng of DNA sample, 5 µl of PCR Master Mix (Fermentas International Inc., Burlington, Canada) and 350 µM of primers specific for *L.* wallacei or *O. fasciatus*. The PCR was performed as follows: initial denaturation of DNA for 5 min at 94°C; 40 amplification cycles each consisting of 30 sec at 94°C, 45 sec at 53°C for both parasite and insect DNA amplification and 30 sec at 72°C; and a final step of 5 min at 72°C for extension of incomplete products. Following PCR, the amplification products were analyzed by electrophoresis in 2% (wt/v) agarose gels that were submitted to ethidium bromide staining and analyzed under ultraviolet light excitation. The expected product sizes were 406 and 176 base pairs for *L. wallacei* and *O. fasciatus*, respectively.

### Interruption of *L. wallacei* transmission by egg surface asepsis

To verify a possible role for cystic forms found on *O. fasciatus* eggshells as infective forms, 450 eggs were collected from the infected colony and submitted to surface asepsis as described above in the topic *Establishment of a parasite-free colony*. Then the eggs were washed in distilled sterile water and dried on sterile filter paper. After asepsis, the eggs were kept in sterile plastic pitchers and the hatched insects maintained in the conditions described for the parental colony, being fed with sterilized water and sunflower seeds until the adult stage. Three pools of five adult insects were randomly collected, dissected in Petri dishes containing sterile PBS and their guts extracted for ruling out infection with *L. wallacei*, by SEM and PCR.

### Scanning electron microscopy

Insect guts were vertically opened in PBS at 4°C before the fixation. Fresh feces were transferred to glass coverslips and immediately fixed or kept at room temperature to dry. The samples were fixed with a solution containing 2.5% glutaraldehyde, 4.0% formaldehyde, 3.7% sucrose and 5 mM CaCl_2_ in 0.1 M cacodylate buffer (pH 7.2), for 2 h at 26°C. After three washes in 0.1 M cacodylate buffer (pH 7.2), samples were dehydrated in increasing concentrations of ethanol, dried using the CO_2_ critical point method in a Balzers apparatus model CDP-20 (Balzers Union, Fürstentum, Liechstenstein), mounted on aluminium stubs with double coated carbon conductive tape and sputtered with gold in a Balzers apparatus model FC-9646. Scanning electron microscopy observations were made under a Jeol JSM-5310 electron microscope.

## Results and Discussion

The relationship between human trypanosomatids and their insect vectors have been thoroughly documented [29]–[34]. On the other hand, the studies on interactions of insect trypanosomatids with their hosts are still incipient [7], [16]–[19], [35], [36]. The occurrence of horizontal transmission of *L. wallacei* by *O. fasciatus* was evident in the present study, corroborating previous data [6]. Here, we collected in our colony at least 50 specimens of the third, fourth and fifth instars, as well as adult insects so as to evaluate the percentage of *L. wallacei*-infected insects observing their intestinal contents by means of optical microscopy. The percentages of infected nymphs were 42, 40 and 39%, for third, fourth and fifth instars, respectively. Strikingly, all of the analyzed adult stage insects were infected (Table 1). Such different rates between nymphs and adults are due to cumulative infection and have been observed both in ticks and insects [37], [38]. Therefore, we concluded that during the insect development, parasites were transmitted between contaminated individuals to parasite-free individuals, or to the environment and then to parasite-free insects.

**Table 1:** Infection rates of insects hatched from eggs laid by *Leptomonas wallacei*-infected *Oncopeltus fasciatus* females.

	Groups (30 insects analyzed per group)										
	Group 1*			Group 2*			Group 3*				
Stage	infected	notinfected	infectionrate (%)	infected	notinfected	infectionrate (%)	infected	notinfected	infectionrate (%)	mean ± SEMof infection rate	
Third instar	17	13	56.7	8	22	26.7	13	17	43.3	42.23±8.67	
Fourth instar	15	15	50.0	9	21	30.0	12	18	40.0	40.0±5.77	
Fifth instar	11	19	36.7	11	19	36.7	13	17	43.3	38.9±2.2	
Adult	30	0	100.0	30	0	100.0	30	0	100.0	100±0	

*Groups 1–3 are biological replicates.

**Figure 1:**
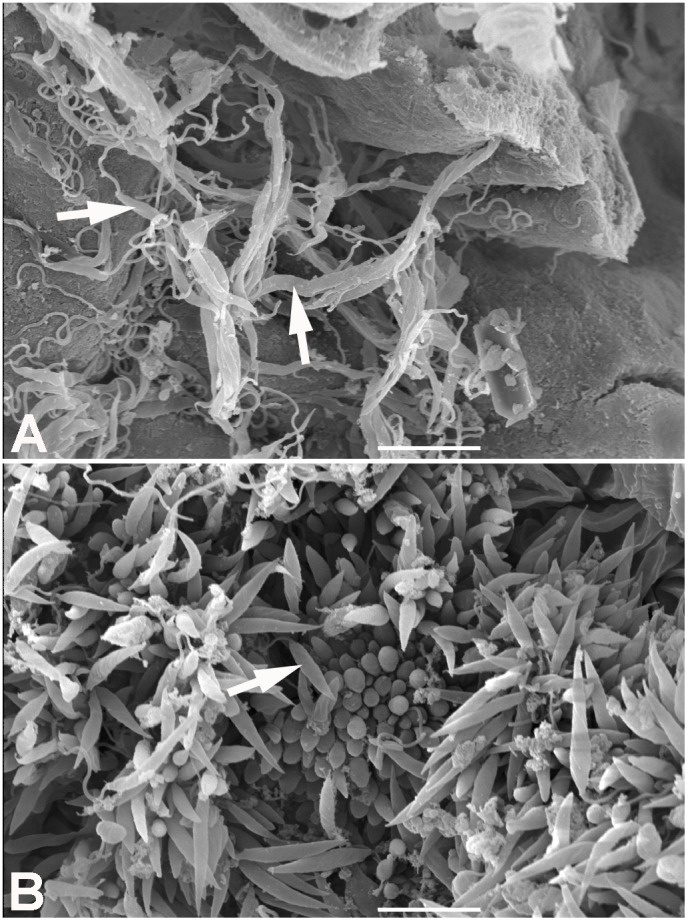
Scanning electron microscopy of *Oncopeltus fasciatus* guts infected with *Leptomonas wallacei*. (A) Midgut of *L. wallacei*-infected *O. fasciatus*. The arrows indicate large numbers of parasites near the midgut wall. (B) Hindgut of *L. wallacei*-infected *O. fasciatus*. The image shows a massive presence of flagellates. Most of those are attached to the intestinal wall of the hindgut by their flagella, so only their slender bodies can be seen. One of the short-sized flagellates can be seen in the lumen (arrow). Bars = 10 µm.

**Figure 2:**
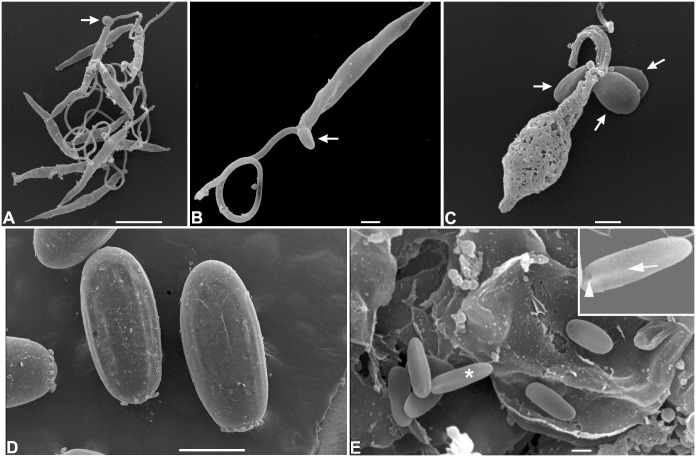
Scanning electron micrograph of feces and eggs of *Oncopeltus fasciatus* infected with *Leptomonas wallacei.* (A) Promastigote forms of *L. wallacei* in fresh feces showing typical characteristics of live cells and cystic forms (arrow). Bar = 5 µm. (B) High magnification of a promastigote form showing one cystic form (arrow) near to its flagella. Bar = 1 µm. (C) Promastigote form present in the naturally dried feces showing extensive membrane damage and three cystic forms. Note that the cystic forms (arrows) do not show any surface damage. Bar = 1 µm. (D) Low magnification of the surface of the eggs. Bar = 500 µm. (E) High magnification of egg surface showing typical cystic forms of *L. wallacei* (arrows). Bar = 1 µm.

In order to detect the infective forms of *L. wallacei*, we analyzed the opened guts of adult insects, feces and eggs collected at the insect colony by means of scanning electron microscopy (SEM). We observed many promastigote forms in the lumen of the midgut (Fig. 1A, arrows). In the hindgut, a massive presence of promastigotes was also evident, most of which were attached to the intestinal wall of the hindgut by the flagella, so that only the slender bodies could be seen (Fig. 1B). The arrow in figure 1B indicates a short-sized flagellate that can be seen in the lumen (Fig. 1B, arrow). We also observed promastigote forms in fresh feces (Fig. 2A), some of which showed cystic forms in association with their flagella, near to the cell body (Fig. 2B). In dried feces there were promastigotes showing extensive cell membrane damage, while the cystic forms attached to them seemed intact, without any visible surface damage (Fig. 2C). Coprophagy among phytophagous and wood-feeding insects seems likely to have been positively selected throughout evolution, since this habit greatly facilitates the transmission of cellulose degrading gut microbiota between these insects [39]. Horizontal transmission by ingestion of parasites present in the insect feces, including cystic forms, has been extensively studied in the interaction between the trypanosomatid *Blastocrithidia triatomae* and the hematofagous insect *Triatoma infestans*, as well as other triatomines [27], [35], [36]. Along with cystic forms, live promastigote forms were observed in fresh feces, so we speculated that horizontal transmission takes place mostly by ingestion of these contaminated feces, which has already been observed for the trypanosomatid *Crithidia bombi* within a population of its natural host, bumblebees of the genus *Bombus* [21].

**Figure 3:**
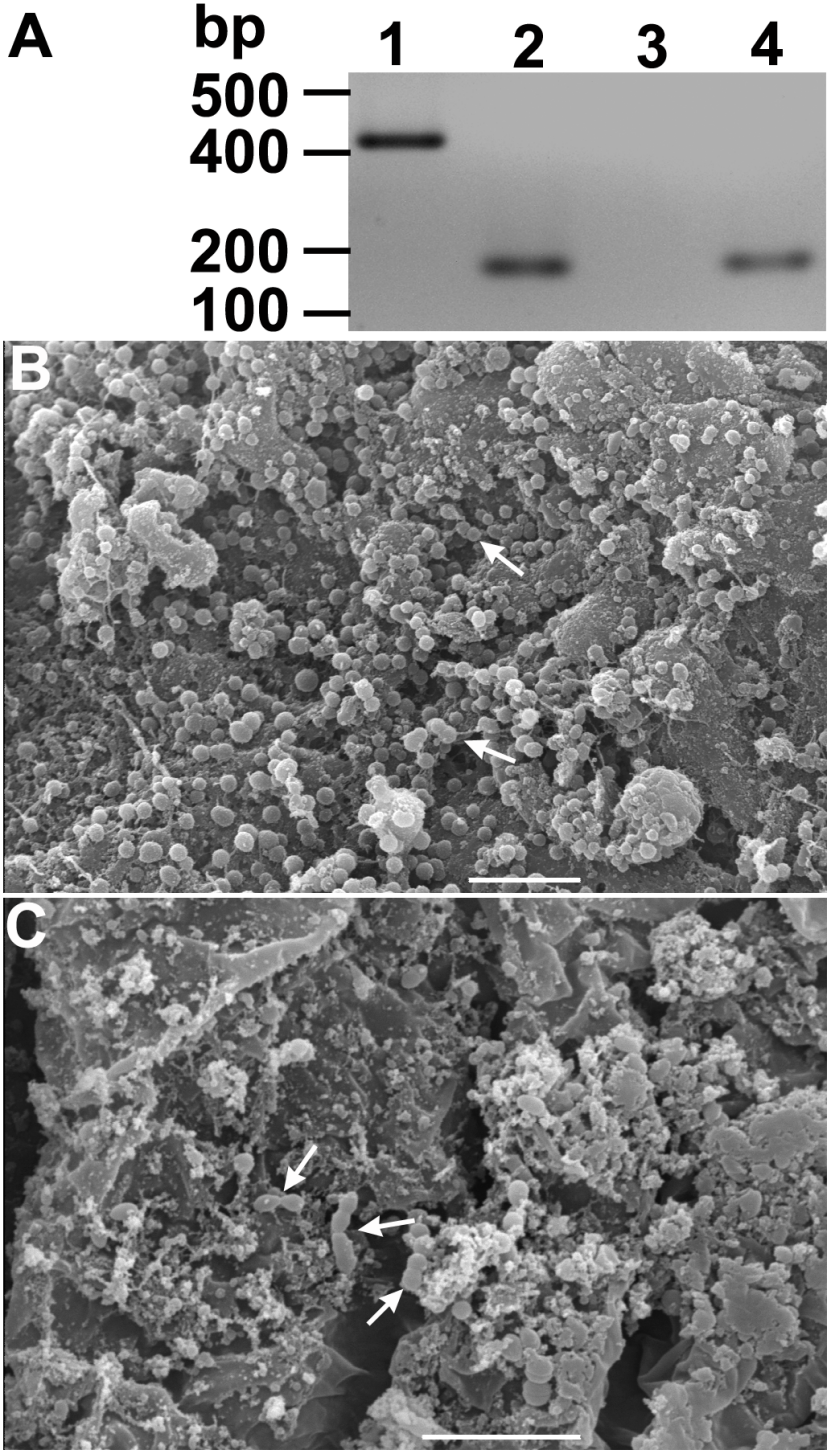
*Oncopeltus fasciatus* hatched from eggs submitted to surface asepsis were *Leptomonas wallacei*-free. (A) Representative gel electrophoresis of PCR-amplified DNA samples extracted from eggs and whole insect guts. Lane 1- The DNA extracted from an axenic culture of *L. wallacei* was amplified with primers specific for parasite detection. Lane 2- Sample of pooled DNA extracted from eggs collected at the infected colony and submitted to surface asepsis was concomitantly amplified with primers specific for parasite or insect DNA detection. Lanes 3 and 4- Sample of pooled DNA, extracted from 3 pools of five insect guts of insects hatched from eggs submitted to asepsis, was PCR-amplified with primers specific for parasite (lane 3) or insect DNA (lane 4) detection, respectively. On the left, the positions of molecular size markers are shown in base pairs. The figure represents a negative image of the gel. (B and C) Scanning electron microscopy of *O. fasciatus* midgut and hindgut, respectively. The micrographs show the presence of bacteria (arrows) but absence of parasites. Bars = 10 µm.

The characteristic cystic forms of *L. wallacei* were observed on the surface of eggs, while promastigotes were never observed there [6]. These cysts showed an ovoid ellipse shape with the expected body size (approximately 3 µm in length), slight body torsion and a small invagination in only one side of each cyst (Fig. 2D and 2E). These findings showed that the cystic forms of the parasites, which most likely contaminate the eggshells during oviposition, may act as infective forms if ingested by the newly hatched nymphs. Similar results have been described for moth eggs contaminated with microsporidian spores [20]. Considering that Porter [26] had suggested transovarial transmission of the trypanosomatid *Crithidia guerridis* in a waterbug *Gerris paludum* population, we searched for the presence of parasite DNA in the eggs collected from the infected colony that were submitted or not to surface asepsis. Total DNA was extracted from whole egg homogenates and PCR-amplified using primers specific for parasite DNA detection. No parasite DNA was found in eggs that were previously treated with sodium hypochlorite, which discarded the possibility of transovarial transmission in our system (Fig. 3A).

The transovum transmission strategy depends on the parasite ability to persist in the environment until egg hatching. Some invertebrate trypanosomatids, including other species of the genus *Leptomonas*, in addition to *L. wallacei*, develop resistant cysts that enable them to persist in the environment for a long time [5], [6], [40], [41]. In order to investigate the remaining possibility of transovum vertical transmission of *L. wallacei*, we isolated eggs from the infected colony and observed newborn nymphs probing and feeding on egg remnants (data not shown). This nymph behavior led us to hypothesize that the cysts of *L. wallacei* that were observed on the eggshells of *O. fasciatus* (Fig. 2E) could be the resistant and infective parasite forms. In addition, we speculated that egg surface contamination occurred during the oviposition because of the contact of eggs with female feces, due to the proximity of the ovipositor with the anus.

Some phytophagous insects eat food sources other than plants, due to the need of a high-protein diet. A newly-hatched nymphs often consume the remainder of their eggshell because the sucking mouthparts of first to third instar nymphs are usually still too small to perforate plants [42], [43]. To investigate the possibility that nymphs naturally acquire infection through the ingestion of cystic forms present on the eggshells after the oviposition, we performed the asepsis of the surface of the insect eggs using sodium hypochlorite [44]. When the insects that hatched from the treated eggs turned into adults, their guts were analyzed by PCR and SEM for the presence of parasites. All the DNA samples extracted from guts of insects developed from eggs treated with sodium hypochlorite were negative for the presence of *L. wallacei*, when tested by PCR (Fig. 3A). In addition, in contrast to the abundant presence of flagellates in the guts of naturally infected insects, only bacteria were observed in the guts of insects that hatched from treated eggs (Fig. 3B and 3C). Therefore, we conclude that transmission of *L. wallacei* from female *O. fasciatus* to the offspring was interrupted by the elimination of *L. wallacei* cystic forms, which usually contaminate the surface of the eggs. Similarly, hypochlorite-treated eggs from the European pine moth, *Rhyacionia buoliana*, gave rise to parasite-free larvae, without any significant decrease in egg hatch. Those larvae could also be aseptically maintained in sterile plastic containers [44].

**Figure 4:**
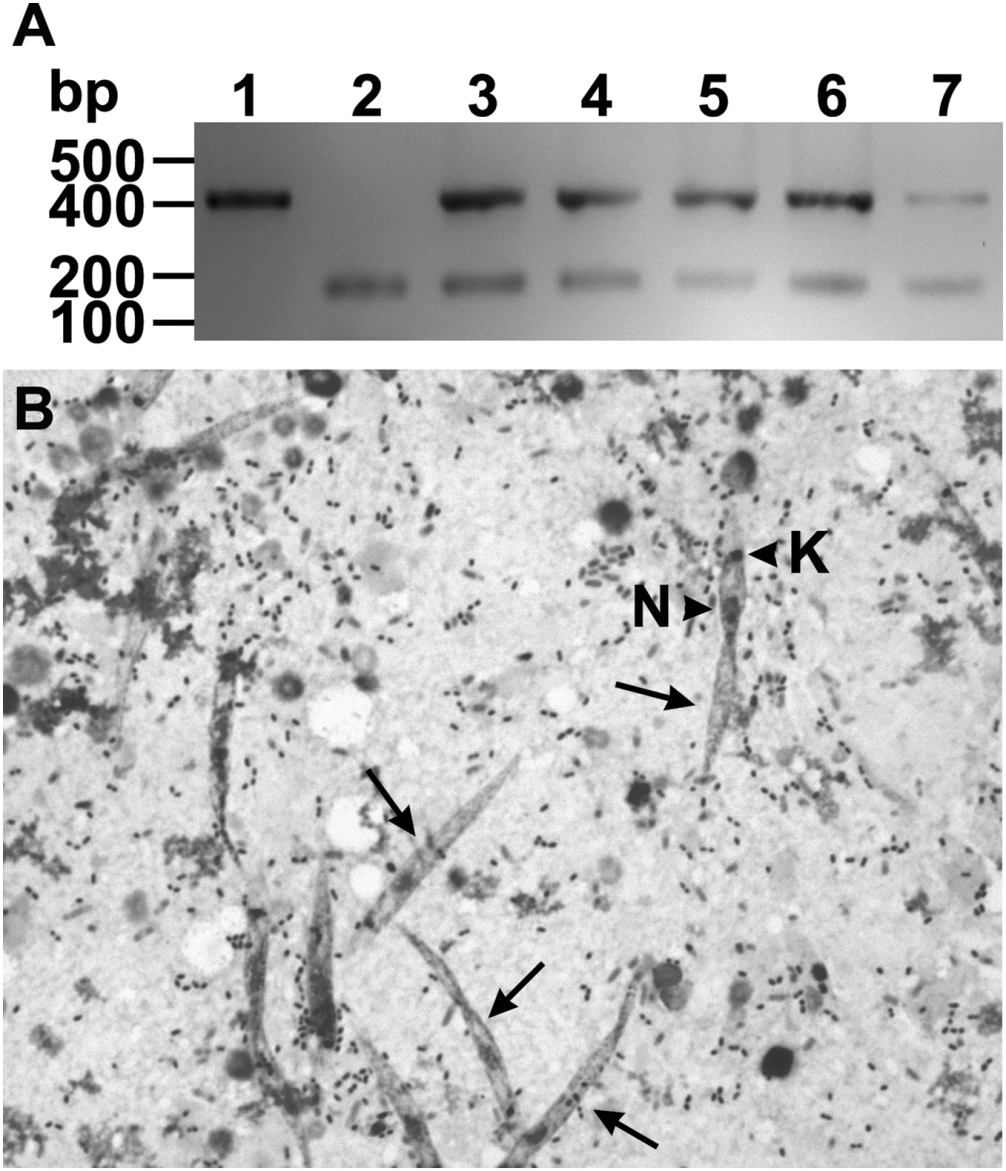
Detection of parasites after experimental transovum transmission of *Leptomonas wallacei* parasites by *Oncopeltus fasciatus*. (A) Detection of parasite infection by PCR. Lane 1- The DNA extracted from an axenic culture of *L. wallacei* was amplified with primers specific for parasite detection. Lane 2- A pool of DNA samples extracted from gut of parasite-free insects was concomitantly amplified with primers specific for parasite and insect DNA detection. Lanes 3–7- Representative DNA samples extracted from guts of insects that fed on sunflower seeds contaminated with eggshels collected from infected colony were concomitantly amplified with primers specific for parasite and insect DNA detection. On the left, the positions of molecular size markers are shown in base pairs. The figure represents a negative image of the gel. (B) Detection of parasite infection by optical microscopy. The representative micrograph shows Giemsa-stained parasites (arrows) in the gut contents of newly infected insects. The arrowheads indicate the nucleus (N) and kinetoplast (K) of the parasite. Magnification = 400 x.

To confirm our hypothesis that nymphs acquire infection by ingesting parasite forms present in the shell of hatched eggs, we added mechanically broken eggs, laid by infected females, to previously sterilized sunflower seeds. These seeds were then offered as the only source of food to parasite-free adult insects. After two weeks, the alimentary tract of these insects were dissected and the parasite infection investigated by optical microscopy and PCR, using primers specific for the detection of parasite DNA. All DNA samples that were extracted from intact guts of these insects were positive for the presence of *L. wallacei* DNA (Fig. 4A). In addition, mobile promastigote forms were found in the digestive tube of these insects (Fig. 4B).

**Figure 5:**
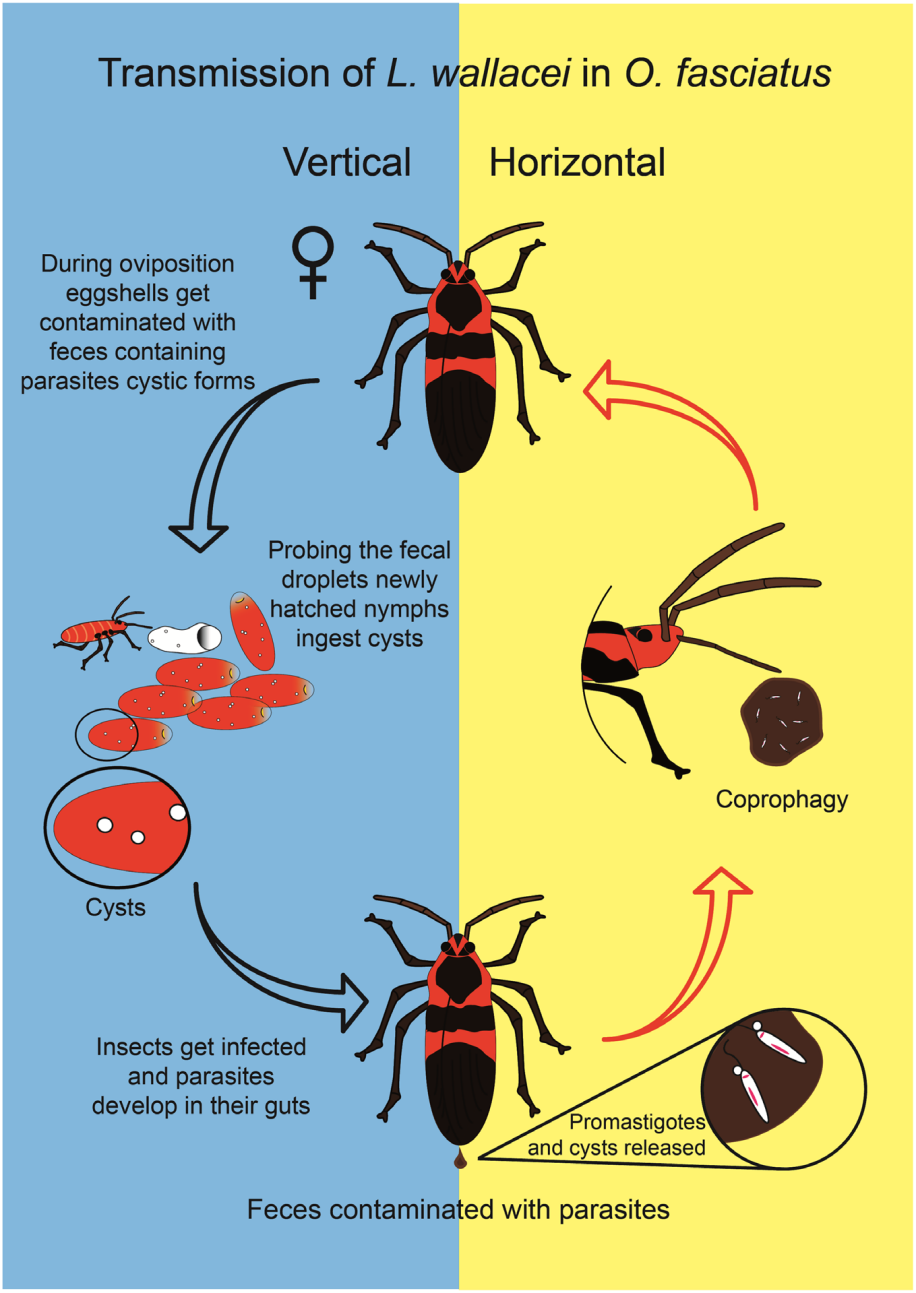
Horizontal and vertical transmission of *Leptomonas wallacei* by *Oncopeltus fasciatus*. The insects get infected by feeding on feces contaminated with parasites. *L. wallacei* produces cystic forms, which are present in the insect guts; the feces contaminate the eggshells. Newly hatched nymphs feed on egg remnants and acquire infection. The cartoon represents a model of transmission based upon previous data and the results shown here.

Along the course of evolution parasites have adapted in order to persist within the host populations [25], [26],[45],[46]. Trypanosomatids are amazingly successful parasites that can be found in all classes of vertebrates, several invertebrates, plants and other protozoa [2], [3]. Also, trypanosomatids can be themselves hosts of endosymbiotic bacteria [47], viruses [48]–[50] or both [50]. Not surprisingly, trypanosomatids harbor “foreign” genetic material, probably originated from plants, bacteria and virus, integrated in their genomes [49], [51], [52] or solely within virus-like particles [50], [52]. Intriguingly, these viruses play a major role in virulence and metastasis of the South American subgenus of the *Leishmania* parasite, *L.* (*Viannia*) [53]. Studying the relationships of trypanosomatids with their hosts is of upmost relevance for better understanding the uniqueness of these parasites. This is particularly true for monoxenous trypanosomatids, which are still poorly understood [54]. Transovum transmission is a strategy shown by different parasites, supposedly with low cost to the host fitness [22], [55], [56]. Even though vertical transmission has been speculated [27], [35], trypanosomatids have only been described as horizontally transmitted by insects. The present study is the first experimental demonstration that vertical (transovum) transmission takes place in the interaction of *O. fasciatus* with *L. wallacei*, a process driven by the contamination of eggshells with resistant cystic parasite forms, which are ingested by the nymphs after hatching. The data described in this study are summarized in a cartoon that shows the horizontal and vertical modes of transmission of *L. wallacei* in *O. fasciatus* (Fig. 5). Based on our findings we hypothesize that, in addition to horizontal transmission, vertical transmission is a feature of the *O. fasciatus*-*L. wallacei* relationship may play an important role in the maintenance of the parasite within its host population.
